# Nod factors potentiate auxin signaling for transcriptional regulation and lateral root formation in *Medicago truncatula*

**DOI:** 10.1093/jxb/erw474

**Published:** 2017-01-10

**Authors:** Violaine Herrbach, Ximena Chirinos, David Rengel, Kokoévi Agbevenou, Rémy Vincent, Stéphanie Pateyron, Stéphanie Huguet, Sandrine Balzergue, Asher Pasha, Nicholas Provart, Clare Gough, Sandra Bensmihen

**Affiliations:** 1 LIPM, Université de Toulouse, INRA, CNRS, Castanet-Tolosan, France; 2 POPS (transcriptOmic Platform of IPS2) Platform, Institute of Plant Sciences Paris Saclay (IPS2), CNRS, INRA, Université Paris-Sud, Université Evry, Université Paris-Saclay, Orsay, France; 3 Institute of Plant Sciences Paris-Saclay IPS2, Paris Diderot, Sorbonne Paris-Cité, Orsay, France; 4 Department of Cell & Systems Biology/ Centre for the Analysis of Genome Evolution and Function, University of Toronto, Toronto, Canada

**Keywords:** Auxin, ethylene, lateral root, lateral root inducible system (LRIS), lipo-chitooligosaccharides (LCOs), NimbleGen arrays, Nod factors, symbiosis, transcriptome

## Abstract

Nodulation (Nod) factors (NFs) are symbiotic molecules produced by rhizobia that are essential for establishment of the rhizobium–legume endosymbiosis. Purified NFs can stimulate lateral root formation (LRF) in *Medicago truncatula*, but little is known about the molecular mechanisms involved. Using a combination of reporter constructs, pharmacological and genetic approaches, we show that NFs act on early steps of LRF in *M. truncatula*, independently of the ethylene signaling pathway and of the cytokinin receptor *MtCRE1*, but in interaction with auxin. We conducted a whole-genome transcriptomic study upon NF and/or auxin treatments, using a lateral root inducible system adapted for *M. truncatula*. This revealed a large overlap between NF and auxin signaling and, more interestingly, synergistic interactions between these molecules. Three groups showing interaction effects were defined: group 1 contained more than 1500 genes responding specifically to the combinatorial treatment of NFs and auxin; group 2 comprised auxin-regulated genes whose expression was enhanced or antagonized by NFs; and in group 3 the expression of NF regulated genes was antagonized by auxin. Groups 1 and 2 were enriched in signaling and metabolic functions, which highlights important crosstalk between NF and auxin signaling for both developmental and symbiotic processes.

## Teaser

Using both pharmacological and transcriptomic approaches, it is shown that Nod factors and auxin interact synergistically to control lateral root formation and gene regulatory responses.

## Introduction

Legume plants have the ability to interact with soil bacteria named rhizobia to establish the rhizobium–legume (RL) symbiosis. Rhizobia are hosted in specific root organs called nodules, where they fix atmospheric nitrogen. This symbiosis provides the plant with nitrogen compounds (ammonium) and the plant provides the bacteria with carbon sources. The efficiency of this interaction relies for a great part on the massive intracellular infection of plant cells by the bacteria and the protective structure of the nodule.

Nod factors (NFs) are lipo-chitooligosaccharide molecules produced by rhizobia in response to flavonoids present in root exudates. NFs are essential for the onset of the RL symbiotic interaction, for host specificity, and for symbiosis maintenance (Dénarié *et al.*, 1996). Genetic pathways governing NF perception and signaling are now quite well understood. NFs are perceived by receptor-like kinases from the Lysin-motif family, and a forward genetic approach isolated the *NOD FACTOR PERCEPTION* (NFP) gene in *Medicago truncatula* (Amor *et al.*, 2003) that encodes the major NF receptor protein. Downstream of this receptor are signaling components that are required for both the RL and the arbuscular mycorrhizal endosymbioses, the so called ‘common symbiosis signaling pathway’ (CSSP) (Catoira *et al.*, 2000).

On top of their role in the establishment of the RL symbiosis, purified NFs also stimulate lateral root formation (LRF) in *M. truncatula* and this response is dependent on the CSSP (Olah *et al.*, 2005). However, this effect of NFs has only been quantified on emerged lateral roots (LRs) and it is not known how NF application on *M. truncatula* triggers LRF. We have recently shown (Herrbach *et al.*, 2014) that LRF in *M. truncatula* is more complex than in Arabidopsis, involving cell divisions in several inner-root tissues. As in Arabidopsis, LRF in *M. truncatula* starts in the pericycle but, in contrast to Arabidopsis, endodermal cell divisions and the innermost cortical cell layer also contribute to the formation of the LR primordium (LRP). This makes the onset of LRF quite similar to that of nodule organogenesis in *M. truncatula*, with similar pericycle and endodermal cell divisions except that, in the case of nodule development, the major contribution to the new organ is from cortical cell divisions (Xiao *et al.*, 2014).

Another similarity between nodule and LR development is their control by hormones. Indeed, most of the major phytohormones such as auxin, cytokinins (CK), ethylene, abscisic acid (ABA), and gibberellins (GA) control both LRF and nodule development in legumes (Bensmihen, 2015). Interestingly, some hormones control these two processes in a similar (positive or negative) way, whereas others play opposite roles. For instance, auxin promotes both LR and nodule development whereas CKs promote nodule development but inhibit LRF, since knock-down of the cytokinin receptor *MtCRE1* produces fewer nodules but more LRs (Gonzalez-Rizzo *et al.*, 2006). Also of interest is the fact that some phytohormones such as ABA have opposite effects on LRF when comparing legumes and non-legumes (Liang and Harris, 2005), which suggests that legumes could have evolved different sensitivities to some phytohormones to control the development of different root organs. Some mutants in hormonal pathways have been found in nodulation screens but their root phenotypes have been poorly investigated. This is the case for the *EIN2* mutant *Mtsickle* (*Mtskl*) that forms more nodules and has a very rapid primary root growth (Penmetsa *et al.*, 2008).

In this work, we have addressed the effect of NFs on LRF in *M. truncatula*. Using a combination of reporter constructs, pharmacological and genetic analyses, we have addressed the stage of LRF targeted by NFs and investigated crosstalk between NF action on LRF and hormonal pathways. Given the very early action of NFs on LRF that we found and its synergistic interaction with auxin, we subsequently adapted a pre-existing method to synchronize and enrich early events of pericycle cell reactivation , allowing us to perform a transcriptomic analysis following NF, auxin, or auxin+NF treatments. Very interestingly, we found that NF and auxin signaling interact at the level of gene regulation in different manners, including a large and novel synergistic manner. These data shed new light on the crosstalk and overlaps of NF and auxin signaling, which are likely to intervene in a variety of symbiotic and/or developmental pathways.

## Materials and methods

### Plant material and treatments

Seedlings of *Medicago truncatula* cv Jemalong A17 (wild-type), *Mtcre1-1* (Plet *et al.*, 2011), *Mtskl* (Penmetsa and Cook, 1997), *Mtnfp-2* (Arrighi *et al.*, 2006) mutants, and DR5:GUS or DR5:VENUS-N7 lines (Herrbach *et al.*, 2014) were grown as described in Herrbach *et al.* (2014).

For hormonal treatments, aminocyclopropane-1-carboxylic acid (ACC, Sigma) was used from a 10^‒2^ M stock solution dissolved in water; naphthaleneacetic acid (NAA) was prepared as described in Herrbach *et al.* (2014); ABA treatment was performed as described in Gonzalez *et al.* (2015); and naphthylphtalamic acid (NPA, Duchefa) was used from a 10^‒2^ M stock dissolved in DMSO.

*Sinorhizobium meliloti* NFs were used from a 10^‒3^ M stock solution dissolved in 50% acetonitrile/50% water.

For RNA extraction 2.5-cm long root segments were harvested 1 mm above the root tip and frozen in liquid nitrogen. Thirty plants per treatment and three biological repeats were used for each condition.

### RNA preparation, NimbleGen arrays and BioMark Q-PCR

Total RNA was extracted using the Qiagen RNeasy Plant mini kit (Qiagen). RNA quality was assessed using the Agilent 2100 BioAnalyzer technology (Agilent technologies). For NimbleGen arrays, RNA samples were treated at the time of extraction by the Qiagen RNase-Free DNase, following the manufacturer’s instructions. A sample of 1 µg of total RNA was sent to the POPS platform for labelling and hybridization (http://www.ips2.u-psud.fr/spip.php?article57). Labeling of cRNAs with Cy3-dUTP or Cy5-dUTP (Perkin-Elmer-NEN Life Science Products) and competitive hybridization to slides were performed as described in Lurin *et al.* (2004) (see below). The Medicago arrays used were based on Roche-NimbleGen technology. A single microarray slide contains 12 chambers, each containing 249 087 long primers representing 83 029 probes corresponding to transcribed regions of the *M. truncatula* genome and 39 403 *Medicago truncatula* coding regions with an Mt4.0 identifier. Each long primer is triplicated for robust analysis.

For Q-PCR experiments, RNA samples were treated using Perfecta DNaseI (QuantaBioSciences), and 1 µg of total RNA was used for reverse transcription using the qScript cDNA SuperMix (QuantaBioSciences), following the supplier’s instructions. Nanoliter high-throughput quantitative PCR (Morrison *et al.*, 2006) was used for validation of 60 genes (using six reference genes) as described in Camps *et al.* (2015). Primers are listed in Table S1 available at the Dryad Digital Repository, http://dx.doi.org/10.5061/dryad.s43c7.

All the original microarray data are deposited in the Gene Expression Omnibus (http://www.ncbi.nlm.nih.gov/geo/) datasets (GSE74099) and at CATdb (Gagnot *et al.*, 2008) (http://tools.ips2.u-psud.fr/CATdb/; Project: 12PLEX_MED_2013-04) according to the ‘Minimum Information About a Microarray Experiment’ standards.

### Data analysis

All inferential and descriptive statistical analyses were carried out in the R environment. More specifically, microarray differential analysis (see below) and non-parametric tests were performed using the limma and PMCMR packages, respectively.

#### Nimblegen arrays and statistical analysis

Experiments were designed with the statistics group of the Institute of Plant Sciences Paris Saclay (IPS2). The following competitive hybridizations were performed: NAA treated versus control, NF treated versus control, NF+NAA treated versus control, and NF+NAA treated versus NAA treated. For each comparison, one technical replicate with fluorochrome reversal including three biological replicates was performed (i.e. two dye-switch hybridizations per comparison). Two-micron scanning was performed with an InnoScan900 scanner (InnopsysR, Carbonne, France) and raw data were extracted using MapixR software (InnopsysR, Carbonne, France).

For each array, the raw data comprised the logarithm of median feature pixel intensity at wavelengths 635 nm (red) and 532 nm (green). For each array, a global intensity-dependent normalization using the LOESS procedure (Yang *et al.*, 2002) was performed to correct for dye bias. The differential analysis is based on log-ratio averaging over the duplicate probes and over the technical replicates. Hence the numbers of available data for each gene equals the number of biological replicates and are used to calculate the moderated *t*-test (Smyth, 2004).

Under the null hypothesis, no evidence that the specific variances vary between probes is highlighted by limma and consequently the moderated *t*-statistic is assumed to follow a standard normal distribution.

To control the false discovery rate, adjusted *P*-values found using the optimized FDR approach of Storey and Tibshirani (2003) were calculated. We considered probes with an adjusted *P*-value ≤0.05 as being differentially expressed. 

The function SqueezeVar of the library limma was used to smooth the specific variances by computing empirical Bayes posterior means. The library kerfdr was used to calculate the adjusted *P*-values.

#### MapMan and Medicago Classification SuperViewer analysis

The MapMan software (http://mapman.gabipd.org/web/guest/mapman version 3.5.1) (Thimm *et al.*, 2004; Usadel *et al.*, 2005) was used for functional classification of genes with an affymetrix probeset. Functional GO enrichment was performed using the Classification SuperViewer tool from the University of Toronto adapted for *Medicago truncatula* Mt4.0 (http://bar.utoronto.ca/ntools/cgi-bin/ntools_classification_superviewer_medicago.cgi) to classify sets of *Medicago* genes according to their functions. The new tool is based on the BAR Arabidopsis Classification SuperViewer framework (Provart and Zhu, 2003) and is called the Medicago Classification SuperViewer (http://bar.utoronto.ca/ntools/cgi-bin/ntools_classification_superviewer_medicago.cgi). To develop this tool, we obtained GO classifications for *Medicago truncatula* genes from two different sources: UniProt, ftp://ftp.ebi.ac.uk/pub/databases/GO/goa/UNIPROT/goa_uniprot_all.gaf.gz, and agriGo, http://bioinfo.cau.edu.cn/agriGO/download/item2term_61 (Du *et al.*, 2010). GO terms and their relationships with one another were parsed from Plant GO (http://www.geneontology.org/ontology/go.obo), and GO Slim term categories based on the most recent GO Slim mappings provided by TAIR (http://www.arabidopsis.org) were used to create the appropriate GO Slim mappings for Medicago GO terms. Go Slim mappings are based on Berardini *et al.* (2004), where approximately 15 most-relevant GO Slim categories per GO aspect (molecular function, biological process, cellular component) were defined. Similar to TAIR GO Slim, we allowed more than one GO Slim term for a given *Medicago* gene in cases where a GO term has multiple parents. The data for Medicago Classification SuperViewer were added to a MySQL database tables on the BAR, and the existing Classification SuperViewer framework was used to build the Medicago Classification SuperViewer using the new database tables. Both UniProt and agriGO data are available in Medicago Classification SuperViewer for Gene Ontology enrichment tests of a user-specified gene set. The UniProt GO dataset is the default option.

### Histochemical and microscopic analysis

GUS staining and root sections were performed as described in Herrbach *et al.* (2014).

### Venn diagrams

Venn diagrams were created using the web application VENNY, an interactive tool for comparing lists with Venn diagrams (http://bioinfogp.cnb.csic.es/tools/venny/index.html).

## Results

### NFs act on early stages of LRF

**Fig. 1.**
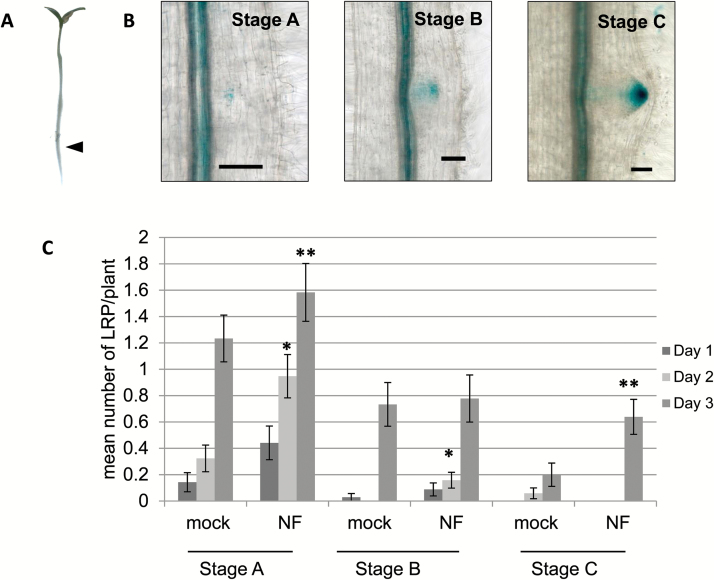
Enrichment in pre-emergence LR stages following local NF application. (A) NFs at 10^‒7^ M were applied in an agar cube at the beginning of the differentiation zone (marked by an arrow) of the primary root of *M. truncatula* DR5:GUS seedlings and LR developmental stages were observed at 1, 2, and 3 d after NF application. LRP staging was performed using the DR5:GUS expression profiles as in Gonzalez *et al.* (2015) and is illustrated in (B), where GUS staining appears in blue. (C) Total number of stage A, B, and C LRP were counted each day (as indicated in the key). Significant differences between the means of the LRP stages (on a given day) between the NF- and mock-treated (control) plants as determined by a non-parametric Kruskal–Wallis test are indicated: *P<0.05, **P<0.01. Scale bars are 1 cm in (A) and 50 µm in (B). Data are from two independent experiments with 30 or 38 plants per treatment. Error bars represent the standard error of the mean.

To date, NF effects on LRF have been tested globally with no focus on specific developmental stages preceding LR emergence. Since DR5 reporter lines are convenient tools to follow LR development, we used our DR5:GUS line (Herrbach *et al.*, 2014) to address the effect of NFs prior to LR emergence. For this, we tested local application of NFs on DR5:GUS lines. Agar cubes (8 mm^3^) containing 10^‒7^ M NFs or a solvent control were applied to the beginning of the differentiation zone of the primary root of 4-d-old seedlings (Fig. 1A, arrow). This zone is known to be sensitive to rhizobium for nodulation (Bhuvaneswari *et al.*, 1981) and we have shown that it also corresponds to the LR initiation zone of *M. truncatula* (Herrbach *et al.*, 2014). We harvested and stained DR5:GUS plants for three successive days following local NF application and used DR5:GUS expression patterns to assign stages to LRP development (Fig. 1B), as done in Gonzalez *et al.* (2015). Stage A corresponded to early stages, B to intermediate, and C to late LRP developmental stages, just preceding LR emergence. After 1 d of local NF application, we observed a slight increase in stage A (Fig. 1C). The effect was more visible after 2 d, when we also noted a slight increase in stage B in treated compared to non-treated plants (Fig. 1C). A significant increase in stage A and pre-emergence stage C LRP was seen after 3 d of NF application (Fig. 1C). Altogether, this suggests that NFs can act locally and at early stages of LRF to stimulate the formation and development of new LRP. Moreover, we observed a significant increase in the total number of emerged LRs after 6 d of NF treatment, with a mean of 4.375 (±1.75 SD) in NF treated vs 3.15 (±2.19 SD) in control plants (data not shown).

### Crosstalk between hormonal and NF signaling pathways for LRF

To further understand the action of NFs on LRF in *M. truncatula*, we used a combination of pharmacological and genetic approaches to address possible crosstalk with phytohormone signaling pathways.

#### ABA

**Fig. 2.**
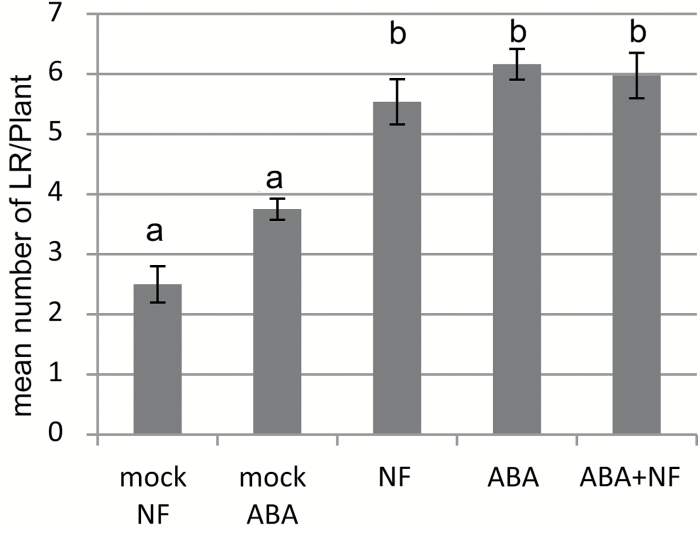
Interaction of ABA and NFs on LRF in *M. truncatula*. Mean number of emerged lateral roots (LRs) observed after 8 d on a growth medium containing 10^‒7^ M ABA and/or 10^‒9^ M NFs. Data are from two independent experiments with an average of 50 plants per treatment. Different letters indicate significantly different means as determined by a non-parametric Kruskal–Wallis test (*P*<0.05). Error bars represent the standard error of the mean.

We have recently shown that 10^‒7^ M ABA stimulates intermediate stages of LR development in *M. truncatula* (Gonzalez *et al.*, 2015). We tested the combined effect of 10^‒9^ M NF and 10^‒7^ M ABA on the total number of LRs. As shown in Fig. 2, and as reported in our previous work, we observed a significant effect of ABA on LRF. The NF effect on LRF was not significantly different from that of ABA in terms of total numbers of LRs formed (Fig. 2), and the combined action of NF+ABA did not show any difference compared with the NF alone or ABA treatments.

#### Ethylene

**Fig. 3.**
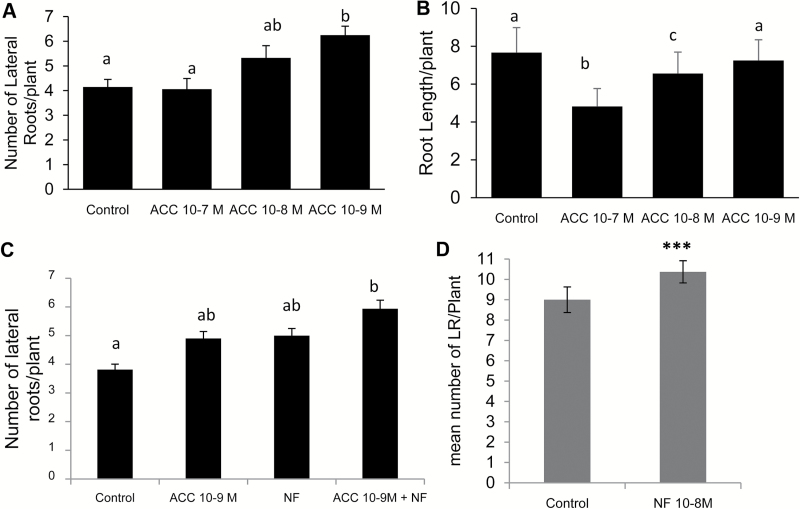
Interaction of ethylene and NF pathways on LRF in *M. truncatula*. Dose–response effects of 1-Aminocyclopropane-l-Carboxylic Acid (ACC) on (A) mean number of emerged lateral roots (LRs) and (B) primary root length of wild-type (A17) seedlings. (C) Influence of 10^‒9^ M ACC and/or 10^‒8^ M NFs on wild-type seedlings. (D) Effect of 10^‒8^ M NFs on *Mtskl* seedlings. Data represent the mean values of three independent experiments. In each experiment 20 seedlings were grown for 13 d. Error bars represent the standard error of the mean. Different letters indicate significantly different means as determined by a non-parametric Kruskal–Wallis test (*P*<0.05). In (D), ***indicates a statistically significant difference at *P*<0.001 as determined by ANOVA.

This phytohormone is known to control both root development and symbiotic interactions (Mohd-Radzman *et al.*, 2013); however, its effect on LRF is poorly described in legumes. To study the effect of ethylene on LRF in *M. truncatula*, we performed a dose–response analysis with the ethylene precursor 1-Aminocyclopropane-l-Carboxylic Acid (ACC). We observed that a low dose of 10^‒9^ M ACC stimulated LRF, whereas 10^‒7^ M ACC did not (Fig. 3A). 10^‒9^ M ACC did not alter primary root length whereas 10^‒7^ M reduced the mean root length of seedlings (Fig. 3B). This resulted in 10^‒7^ ACC having the clearest effect on LR density (Supplementary Fig. S1 available at *JXB* online). 10^‒9^ M ACC and 10^‒8^ M NF seemed comparatively equivalent for LRF stimulation after 13 d (Fig. 3C) and 10^‒8^ NF did not show any effect on primary root growth (data not shown). We used 13 d as the time of treatment in order to be able to study *Mtskl* LRF properly, as LRP emerged later in this mutant. With combined ACC and NF treatments, we observed an additive effect on LRF (Fig. 3C). We further tested the interaction between NFs and ACC on LRF by using the *Mtskl* mutant, which is deficient in ethylene perception (Penmetsa *et al.*, 2008). In untreated conditions, the *Mtskl* mutants displayed more emerged LRs than the wild-type due to very rapid primary root growth, but *Mtskl* LR density was lower than that of the wild-type, although this was not statistically significant (Supplementary Fig. S2). When we grew *Mtskl* seedlings in the presence of ACC they did not show any difference in numbers of LRs and primary root length (Fig. S3), in contrast to the wild-type plants. In contrast, *Mtskl* was still sensitive to NFs for the LRF response (Fig. 3D). NF treatment did not affect *Mtskl* primary root length (Supplementary Fig. S4). Moreover, the NF effect was also visible on the number of non-emerged LRP (visible under a binocular microscope), which were more numerous in NF-treated than in control conditions (Supplementary Fig. S5).

#### Cytokinins (CKs)

**Fig. 4.**
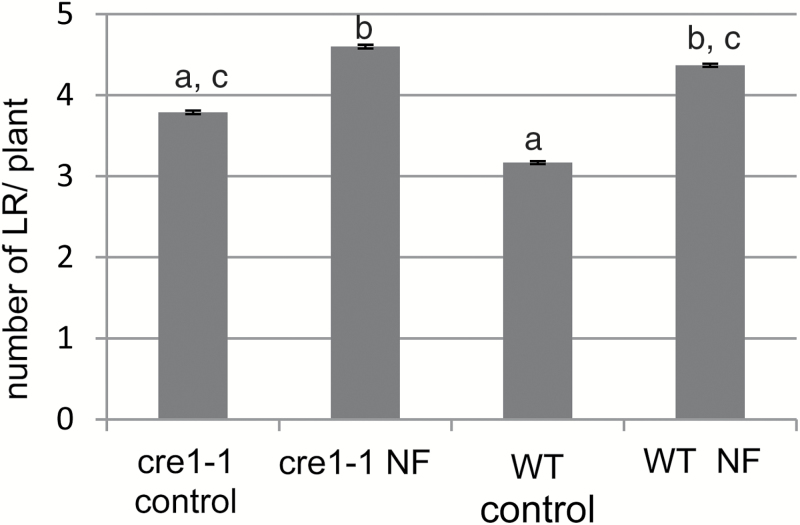
Interaction of cytokinin and NF pathways on LRF in *M. truncatula*. *Mtcre1-1* (cre1-1) and its wild-type sibling (WT) were tested for LRF stimulation by 10^‒8^ M NFs for 8 d. Data are the mean numbers of emerged LRs observed in two independent experiments, with 40 seedlings each. Error bars represent the standard error of the mean. Different letters correspond to significantly different groups as determined by a non-parametric Kruskal–Wallis test (*P*<0.05).

These are also phytohormones with effects on both LRF and nodulation in legumes, with the cytokinin receptor MtCRE1 controlling LRF and nodule development in an opposite manner (Gonzalez-Rizzo *et al.*, 2006). Therefore, we tested the ability of the *Mtcre1-1* mutant (Plet *et al.*, 2011) to respond to NFs for LRF. In our system, *Mtcre1-1* seedlings displayed slightly more LRs than their wild-type siblings, as expected, but were sensitive to NF action for the stimulation of LRF (Fig. 4). No NF effect on the primary root length was observed in the mutant or its wild-type sibling (Supplementary Fig. S6).

#### Auxin

**Fig. 5.**
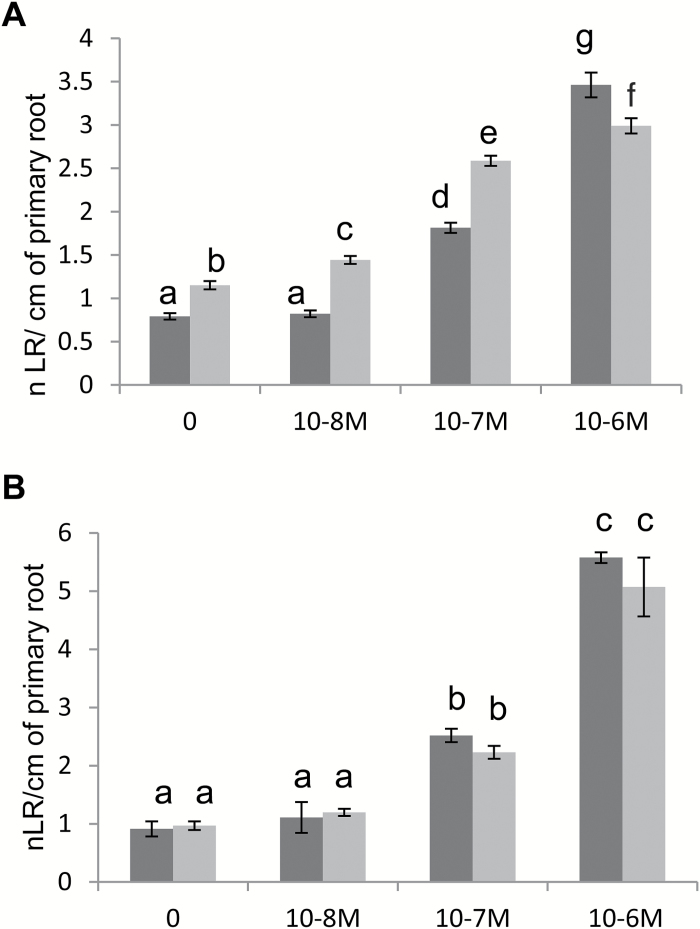
Interaction of auxin and NF pathways on LRF in *M. truncatula*. Dose–response effects of the auxin analog 1-naphthaleneacetic acid (NAA) (10^‒8^ M to 10^‒6^ M) on the LR density (ratio of LRs formed per cm of primary root) of seedlings of (A) wild-type (A17) plants and (B) the NF perception mutant *nfp-2*, in the absence (dark grey) or presence (light grey) of 10^‒8^ M NFs for 8 d. Data are the mean numbers of emerged LRs observed for 38 to 55 seedlings for each treatment. Error bars represent the standard error of the mean. Different letters correspond to significantly different groups as determined by a Kruskal–Wallis test and *post hoc* van Waerden normal scores test for multiple comparisons (with a Benjamini Hodchberg correction) (*P*<0.05). An ANOVA comparing the NF+NAA additive model to a NF*NAA interaction model for LR density, followed by a *post ho*c Tukey test, found a significant difference (i.e non-additivity) with a *P*-value <0.05.

This is the major phytohormone controlling LRF in plants (Overvoorde *et al.*, 2010). We studied the sensitivity of *M. truncatula* seedlings to auxin using different concentrations of the auxin analog 1-naphthaleneacetic acid (NAA). We observed that increasing concentrations of NAA increased LR density (Fig. 5A) as there was both a stimulation of LRF (Supplementary Fig. S7A) and a reduction in primary root length (Fig. S7B). When we combined 10^‒8^ M NF with 10^‒8^ or 10^‒7^ M NAA, we observed an effect on LRF that was significantly more than the additive action of either molecule used separately (Fig. 5A). Interestingly, this synergistic action was not seen on primary root length (Supplementary Fig. S7B). This synergistic interaction was lost in the *nfp-2* mutant, which is completely defective for NF perception, while it retained sensitivity to auxin, indicating that this synergy was dependent on NF perception (Fig. 5B).

### *Lateral root inducible system set-up in* M. truncatula

**Fig. 6.**
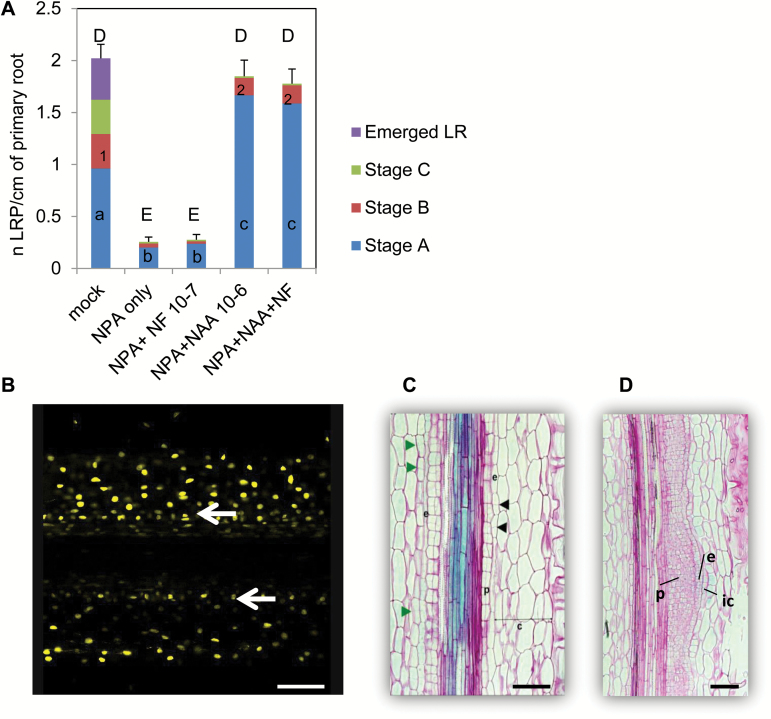
Set-up of the lateral root inducible system (LRIS) in *M. truncatula*. (A) LRP developmental stages observed in DR5:GUS transgenic seedlings grown for 4 d on M medium (mock) or for 2 d on M medium and then transferred to either a 10^‒5^ M 1-N-naphthylphthalamic acid (NPA) medium for 2 d (NPA only), or to NPA for 2 d and then for 2 d on 10^‒7^ M NFs (NPA+NF 10^‒7^) or 10^‒6^ M NAA (NPA+NAA 10^‒6^) or a combination of 10^‒7^ NFs and 10^‒6^ M NAA (NPA+NAA+NF). LR developmental stages are as described in Fig. 1. Data are the mean numbers of LR stages observed for 34 to 54 seedlings for each treatment. Different lower-case letters (a, b, c) correspond to significantly different groups among the stage A LRP, different numbers (1, 2) correspond to significantly different groups among the stage B LRP, and different upper-case letters (D, E) correspond to significantly different groups among total LRP number following an ANOVA and Fisher *post hoc* least-significant difference (LSD) test (*P*<0.05). (B) Confocal microscopy image of a longitudinal section of a DR5:VENUS-N7 transgenic root showing pericycle cell divisions (white arrows) following growth for 2 d on M medium, then a 2-d NPA treatment followed by a 10-h 10^‒6^ M NAA treatment. (C, D) Longitudinal sections of a DR5:GUS seedling grown for 2 d on M medium and then transferred for 2 d on 10^‒5^ M NPA and then for (C) 20 h or (D) 48 h on 10^‒6^ M NAA. Green and black arrowheads highlight anticlinal divisions in the cortex and endodermis, respectively. Abbreviations: p, pericycle; e, endodermis; c, cortex; ic, inner cortex. Sections are 8 µm thick and are counterstained by ruthenium red. Scale bars represent 100 µm.

To further understand the synergistic interaction between NFs and auxin, we decided to set up a transcriptomic approach. Since NFs act on early stages of LRF and interact synergistically with auxin, we used a protocol to synchronize and enrich in the early stages of LRF, adapted from the lateral root inducible system (LRIS) used in Arabidopsis (Himanen *et al.*, 2002). This protocol is based on the inhibition of polar auxin transport by 1-N-naphthylphthalamic acid (NPA) to disturb the endogenous auxin gradient, followed by treatment with a massive dose of an auxin permeant analog (NAA) to restart cell divisions and the LRF program. Using the DR5:GUS line, we showed that all three of the main LRP stages and emerged LRs were present in plants grown for 4 d without any treatment. If seedlings were pre-grown for 2 d and then transferred for 2 d to 10^‒5^ M NPA, we observed an efficient block on development with only a few LRP in stages A and B being visible (Fig. 6A). No further LRP development was visible if we transferred the seedlings for 2 d to 10^‒7^ M NF after the NPA treatment. In contrast, a 2-d treatment with 10^‒6^ M NAA following the 2-d NPA treatment, alone or in combination with NFs, resulted in significantly higher total numbers of LRP in stages A and B (Fig. 6A). We then took longitudinal sections of roots treated for 10, 20, or 48 h with 10^‒6^ M NAA after a 2-d NPA treatment. Using our DR5:VENUS-N7 reporter line, we found that a 10-h NAA treatment corresponded to the onset of the first pericycle periclinal divisions (Fig. 6B). After 20 h of NAA treatment, we observed anticlinal and periclinal divisions in the pericyle and endodermis layers, as well as anticlinal divisions in the inner cortex (Fig. 6C). After 48 h of NAA treatment, we observed more advanced LRP with extended divisions of the pericycle cell layer (Fig. 6D). After 7 d on NAA, emerged LRs were visible all along the primary root (data not shown). This showed that the cell divisions observed during LRIS were similar to those that are characteristic of normal LRF in *M. truncatula*, and that this system was efficient in stimulating LRF in an extended and synchronous manner in *M. truncatula*.

### Transcriptomic approach

**Fig. 7.**
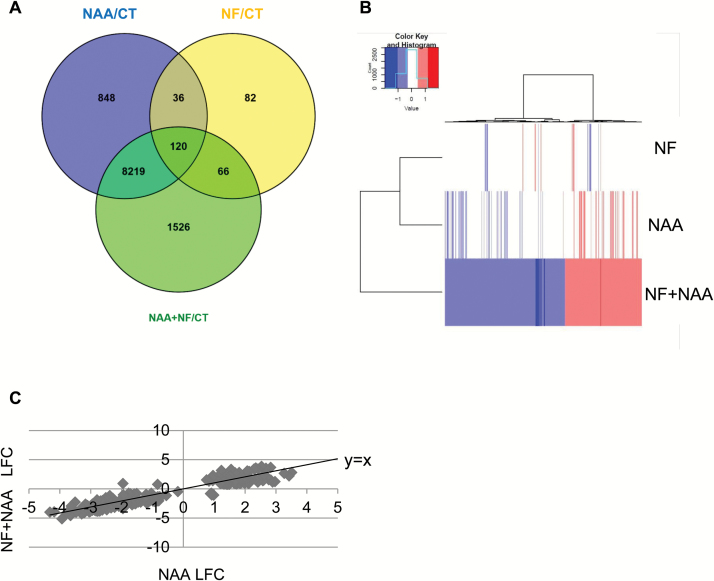
Analysis of the differentially expressed genes (DEGs). Transcriptomic analysis identified 10 897 Mt4.0 DEGs with a Benjamini Hochberg *P*-value ≤0.05 and an absolute Log_2_ fold-change of 0.75 minimum. (A) The Venn diagram represents the distribution of all these DEGs per treatment (NF, Nod factors 10^‒7^ M; NAA, NAA 10^‒6^ M; CT, mock control). (B) A heatmap clustering of the 1526 DEGs found in the NF+NAA/CT synergistic group depending on their expression ratio in the NF, NAA and NF+NAA treatments. Red and blue colors represent up- and down-regulated genes, respectively. (C) Relative expression of the 266 genes from the second synergistic category upon NAA treatment (*x*-axis, Log_2_ FC) and the NF+NAA treatment (*y*-axis, Log_2_ FC). A line representing *y*=*x* is shown to better visualize the shift in response to the NF+NAA condition compared to NAA alone. LFC = Log_2_ fold-change.

Two-day-old *M. truncatula* seedlings were grown for 2 d on 10^‒5^ M NPA medium and then transferred for 10 h onto a medium containing either 10^‒7^ M NF, 10^‒6^ M NAA, 10^‒7^ M NF+10^‒6^ M NAA, or controls with only the same concentration of solvents used for the NF and NAA treatments. Total RNA was extracted and sent to the POPS platform for NimbleGen array analysis. This array represents the most complete array of the *M. truncatula* genome (see Methods). Figure 7 summarizes the number of differentially expressed genes (DEGs) identified for each treatment, using a minimum log_2_ fold-change of ±0.75 and an adjusted *P*-value ≤0.05.

#### NF and auxin signaling overlap

As expected, NAA had a strong effect, with 9223 genes responding to the auxin treatment and 9931 DEGs identified for the NAA+NF treatment compared with control conditions (with 8339 DEGs overlapping, Fig.7A). Among the 9223 DEGs, 4521 were up-regulated and 4702 were down-regulated (see Table S2 at Dryad). Well-known auxin-responsive genes, such as the transcription factors *FEZ* (Medtr5g040420), *SOMBRERO* (Medtr2g062730), *LBD16* (Medtr7g096530), and *PUCHI* (Medtr4g119270), the efflux carrier transporter *PIN3* (Medtr4g084870), and the *GH3.1* auxin-responsive gene (Medtr0035s0150) were up-regulated in both NAA and NAA+NF conditions. Several IAA/AUX, SAUR, and cyclin genes also responded to the auxin treatment (Table S2 at Dryad). Altogether, this suggests that our system was highly responsive to auxin. Despite the NPA pre-treatment, the roots retained sensitivity to NFs, since 304 genes responded to the NF treatment (204 up-regulated and 100 down-regulated, Table S3 at Dryad). Among these genes were the well-known symbiotic marker genes *MtENOD11* (Journet *et al.*, 2001), *MtN1* (Gamas *et al.*, 1998), the U-Box protein *MtPUB1* (Mbengue *et al.*, 2010), and the transcription factor *NSP1* (Smit *et al.*, 2005). Interestingly, approximately half (156/304) of the NF-responsive DEGs were also responsive to NAA. This included the symbiotic genes *MtNSP1*, *MtVAPYRIN* (Murray *et al.*, 2011), and *MtLYK10*. These common target genes of NF and auxin signaling were regulated in both a similar and an opposite manner. For example, *MtLBD16* was up-regulated both by auxin and by NFs, but *MtNSP1* and *MtVAPYRIN* were down-regulated by NAA and up-regulated by NFs. Some hormone-related genes such as *MtIPT5* (Medtr2g022140) and a GA2 oxidase (*GA2Ox1*, Medtr2g019370) were also regulated in an opposite manner by NAA (negative effect) and NFs (positive effect) (Table S3 at Dryad).

In many cases, the action of NFs and NAA was additive, with a fold-change in the NF+NAA treatment that was approximately the sum of the action of NAA and NFs individually (see Tables S2, S3 at Dryad). In some cases, one treatment effect was dominant over the other. For example, the MAPKKK14-like gene (Medtr5g071560) was predominantly regulated by NFs and the *MtLBD16* transcription factor by NAA (Table S3 at Dryad). Altogether, these data suggest a strong convergence and interaction between the NF and auxin signaling pathways, acting via common and/or parallel pathways.

Of the 9931 genes that responded differentially to the NF+NAA treatment compared with control conditions (Fig.7, Table S4 at Dryad), 1526 responded specifically to the combinatorial effect of NF+NAA (616 were up-regulated and 910 down-regulated; Fig.7, Table S5 at Dryad). This first ‘molecular synergistic’ group encompassed symbiotic genes such as *MtNFP* (Medtr5g019040), *MtLYK3* (Medtr5g086130), and *MtNSP2* (Medtr3g072710), as well as hormone-related genes such as the potential ortholog of the auxin biosynthesis gene *AtTAA1* (Medtr5g033520), the auxin influx carrier *MtLAX1* (Medtr5g082220), and a cytokinin oxidase gene (Medtr4g126150, *MtCKX2*) (Table S5 at Dryad). Heatmap clustering of these 1526 genes clearly showed a different response to the combined NF+NAA treatment compared with NF or NAA treatments alone (Fig. 7B), although several genes had a slight (not statistically significant) tendency to respond to NAA treatment alone, suggesting that NFs can potentiate auxin action.

Among the NAA-regulated genes, we wanted to investigate genes for which NF+NAA treatment had a greater effect than NAA only. To identify such genes, we directly compared NF+NAA with NAA-treated roots and found 1038 DEGs (see Table S6 at Dryad). Among these DEGs, 266 were responsive to NAA and NF+NAA but not to NFs compared with control conditions. We called these genes ‘synergistic group 2’ (Table S7 at Dryad). By plotting the expression levels of these genes in the NF+NAA condition compared to NAA, we found 105 genes where NFs antagonized NAA action and 161 genes for which NFs enhanced the NAA effect (Fig. 7C). Among these 266 genes, we found no obvious symbiosis-related genes but many hormone-related genes. For instance, the ACC oxidase *MtACS3* (Medtr5g015020) and the (putative) ABA biosynthetic gene *MtABA4* (Medtr6g025680) were more significantly up-regulated by the NAA+NF treatment compared to the NAA treatment alone and did not respond to the NF treatment alone (Tables S7, S8 at Dryad). Hormone-signaling genes, such as *MtIAA7* (Medtr3g106850) and one potential negative regulator of LRF, the ortholog of *AtPRR7* (Medtr1g067110) (Ruts *et al.*, 2012) were also represented (Tables S7, S8 at Dryad). Expression levels of other hormone-related genes such as the auxin biosynthesis gene *MtTAR2* (Medtr3g077250) and the CK signaling gene *MtRR9* (Medtr4g051330) were also different in the NAA and NF+NAA treatments compared with control conditions, but no differential expression was observed with the direct NAA comparison (see Tables S8, S2 at Dryad). The same was found for the negative regulator of LRF *MtCRA2* (Medtr3g110840) (Huault *et al.*, 2014).

Finally, we looked at the 66 genes responding to both NFs and NF+NAA but not to NAA for genes showing a difference between NF and NF+NAA responsiveness. To do this, we chose an expression level different by at least 30% between the NF and the NF+NAA treatments and found 51 such genes (see Table S8 at Dryad). Most of these genes were up-regulated by NFs and showed a less pronounced induction in the NF+NAA condition. Only two of them (ENOD11 and a Kunitz protease inhibitor, Medtr6g045097) were more highly induced by the NF+NAA combination compared with NFs alone. This suggests that auxin can also negatively interfere with NF signaling.

#### Q-PCR validation

Validation of the arrays was performed using high-throughput Q-PCR analysis of 60 genes showing different expression profiles on three new independent biological repetitions (Supplementary Fig S8). A similar analysis performed using the *nfp-2* mutant indicated that the NF effect on gene expression was lost both for the NF and NF+NAA treatments in *nfp* roots, demonstrating that the NF+NAA genetic interaction is dependent on NF perception (Supplementary Fig. S9).

#### Functional category enrichment in the synergistic groups of genes

**Fig. 8.**
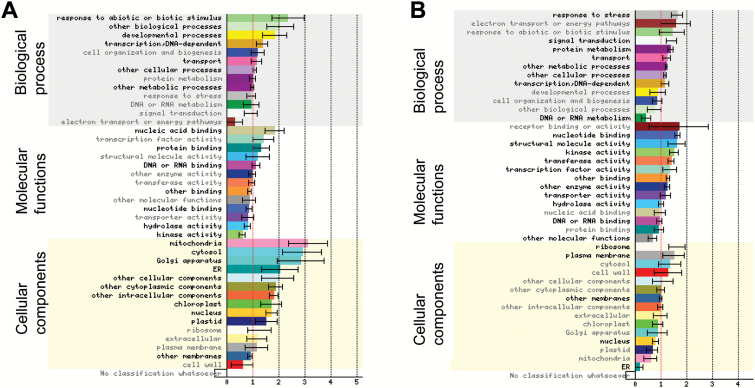
Functional category classification of the up- and down-regulated genes from the first synergistic group. Graphical summary of the gene ontology (GO) classification ranking of the 616 up-regulated (A) and 910 down-regulated (B) genes of the first synergistic group obtained using the Classification SuperViewer tool from http://bar.utoronto.ca adapted to *Medicago truncatula*. The data are the normed frequency of each GO category for the given sets of genes compared to the overall frequency calculated for the Mt4.0 *Medicago truncatula* genome as: (Number_in_Class_input_set_/Number_Classified_input_set_)/(Number_in_Class_reference_set_/Number_Classified_reference_set_). Hence, a frequency above 1 means enrichment and below 1 means under-representation. Errors bars are the standard deviation of the normed frequency calculated by creating 100 gene sets from the input set by random sampling (with repeats allowed, i.e. 100 bootstrapped data sets) and computing the frequency of classification for all of those data sets across all categories. Hypergeometric enrichment tests on the frequencies were performed and GO categories showing significant *P*-values (<0.05) are highlighted in bold. GO categories are displayed for each GO subclass ranked by normed frequency values.

To better understand the relevance of these synergistic regulations, we performed functional enrichment tests using Gene Ontology (GO) categories and the Classification Superviewer interface (CSV, from bar.utoronto.ca) adapted to the Mt4.0 version of the *M. truncatula* genome. Among the 1526 genes in the first synergistic category, 1117 could be classified (Fig. 8). Both up- and down-regulated genes showed a significant enrichment in transcriptional activities (present in ‘transcription-DNA dependent’ and in ‘other cellular processes’ categories) and in ‘transport’ activities (with many ABC transporters in the down-regulated genes). Functions related to phototropism, gravitropism, or fungal responses (‘response to abiotic and biotic stimulus’), SAUR-like genes (‘other biological processes’), and genes related to pollen or root hair development (‘developmental processes’), as well as ‘protein binding’ activities were specifically enriched in up-regulated genes (Fig. 8A). In contrast, disease resistance genes (TIR-NBS-LRR, belonging both to ‘stress response’ and ‘signal transduction’ categories), genes encoding proteins with phosphorylation, dephosphorylation or ubiquitin ligase capacities, as well as ribosomal subunits (represented in ‘protein metabolism’) were specifically enriched in down-regulated genes. Accordingly, enrichment in the cellular localization corresponding to ‘ribosome’ and ‘plasma membrane’ was observed for down-regulated genes whereas many different cellular compartments seemed enriched in the up-regulated ones (Fig. 8). Metabolic and signaling functions represented in ‘other metabolic processes’, ‘nucleotide binding’, ‘other binding’, ‘kinase’, and ‘hydrolase activity’ were under-represented in up-regulated genes but enriched in down-regulated genes (Fig. 8A, B). ‘DNA or RNA binding’, containing many transcription factors (TFs) and genes related to the transcriptional/translational machinery, was enriched in up-regulated genes and under-represented in the down-regulated genes. A more visual representation was obtained using the Mapman software. A total of 1305 genes could be tested, and among them 252 genes could be assigned to the global ‘regulation’ (Supplementary Fig. S10A) and 218 to the ‘biotic stress’ categories (Fig. S10B). A large representation in protein kinases, TFs (71 from different types), and protein degradation pathway components was observed, as well as the presence of several hormone-related genes, more specifically related to auxin and ethylene pathways (Fig. S10). ‘Biotic stress’ genes encompassed a variety of biological functions, including cell wall modification, proteolysis (E3 ligases) as well as secondary metabolism (phenylpropanoids and flavonoids), and signaling components (hormone signaling, TFs, and receptor kinases) (Fig. S10B).

**Fig. 9.**
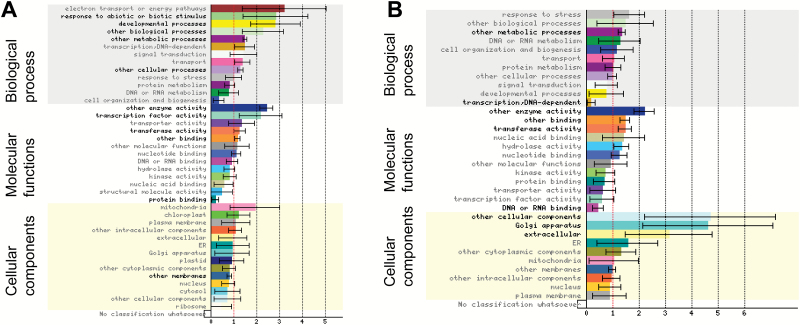
Functional category classification of the positive interaction and negative interaction genes from the second synergistic group. Graphical summary of the gene ontology (GO) classification ranking of the 161 differentially expressed genes (DEGs) from the second synergistic category for which NFs enhanced the NAA effect (positive interaction, A) and the 105 DEGs for which NFs antagonized the NAA effect (negative interaction, B), using the Classification SuperViewer tool from http://bar.utoronto.ca adapted to *Medicago truncatula*. The data are the normed frequency of each GO category for the given sets of genes compared to the overall frequency calculated for the Mt4.0 *Medicago truncatula* genome as: (Number_in_Class_input_set_/Number_Classified_input_set_)/(Number_in_Class_reference_set_/Number_Classified_reference_set_). Hence, a frequency above 1 means enrichment and below 1 means under-representation. Errors bars are the standard deviation of the normed frequency calculated by creating 100 gene sets from the input set by random sampling (with repeats allowed, i.e. 100 bootstrapped data sets) and computing the frequency of classification for all of those data sets across all categories. Hypergeometric enrichment tests on the frequencies were performed and GO categories showing significant *P*-values (<0.05) are highlighted in bold. GO categories are displayed for each GO subclass ranked by normed frequency values.

A similar analysis was conducted on the 266 DEGs from our second synergistic group, separately for the 105 DEGs where NFs counteracted NAA regulation (negative interaction) and for the 161 DEGs where NFs enhanced NAA action (positive interaction). Using the CSV tool, we found enrichment in several metabolic processes and signaling pathways, with several cytochrome p450 enzymes, oxidases, protein kinases, and E3 ligases (represented in ‘other metabolic processes’, ‘other enzyme activity’, and ‘transferase activity’) in both groups. In contrast, TFs and receptor kinases (in ‘other cellular processes’ and ‘transcription factor activity’) were enriched in the positive interaction group but TFs and DNA-interacting proteins were under-represented in the negative interaction group (Fig. 9). ‘Response to abiotic and biotic stimulus’, ‘developmental processes’, and ‘other biological processes’ that were enriched in the positive interaction group (Fig. 9A) contained a few (4–6) genes that were related to light stimulus, SAUR-like genes, and TFs. Using the Mapman software, we observed representation of many general ‘regulation functions’ (TFs and protein kinases) (Supplementary Fig. S11A). Signaling, hormone signaling, and respiratory burst processes were more represented in the positive interaction category while peroxidases, beta glucanases, and secondary metabolism functions were more present for genes showing negative interaction between NFs and NAA (Fig. S11B, C).

## Discussion

### *NFs act early on LRF, independently of the* MtCRE1- *and* MtSKL-*dependent pathways and in synergy with auxin*

NFs are signaling molecules produced by rhizobia that are generally necessary to establish the rhizobium–legume symbiosis (Dénarié *et al.*, 1996). These molecules also induce the formation of new LRs in *M. truncatula* via the CSSP (Olah *et al.*, 2005). However, only emerged LRs have been assessed so far and little is known about how NF signaling impacts on LR developmental pathways. Here, we showed that NFs act on very early stages of LRF, and most probably on LR initiation (LRI) since local application of NFs on the LRI zone of *M. truncatula* resulted in a significant increase of early LRP stages after one day, which was followed by an increase in subsequent LRP stages. However, we cannot rule out later additional effects on LRP development and/or emergence. To test this possibility, it would be interesting to examine the effects of local NF application on older parts of the primary root, where LRP are already initiated. Very interestingly, we observed that NFs act synergistically with auxin specifically on LRF and not on primary root length. This suggests that NFs target local auxin gradients or signaling and do not disrupt the global auxin distribution.

We have also shown that NF action on LRF is independent of key genes controlling CK and ethylene signaling. Since CKs play a negative role on LRF in *M. truncatula* ((Gonzalez-Rizzo *et al.*, 2006; our data), our results show that NFs do not interfere with LRF by simply relieving a CK-controlled negative regulation of LRI. For ethylene, we observed that a similar concentration range as that used in Arabidopsis (Ivanchenko *et al.*, 2008) could enhance LRF in *M. truncatula* and that this stimulation was dependent on *MtSKL*. *Mtskl* seedlings grow very fast and display more LRs than the wild-type, but remain sensitive to NF action. The fact that NFs can increase the number of both emerged and non-emerged LRP in *Mtskl* is consistent with an early action of NFs on LRF, although the precise staging of LRP development in *Mtskl* in the presence or absence of NFs was not assessed here.

As previously described (Liang and Harris, 2005), we observed that ABA had a positive effect on LRF in *M. truncatula* and this positive action seemed not to be additive to NF action. We have previously shown that ABA stimulates intermediate developmental stages of LRF in *M. truncatula* (Gonzalez *et al.*, 2015), so it could well be that NFs affect such stages as well as LRI.

Both ACC and ABA inhibit nodulation in *M. truncatula* and interfere with NF signaling, leading to calcium spiking (Oldroyd *et al.*, 2001; Ding *et al.*, 2008). However, the concentrations used to inhibit NF signaling (10^‒6^ M for ACC and 10^‒3^ M for ABA) are higher than those used here, so the lack of any combinatorial effects observed for LRF with ABA and NFs is unlikely to be due to a block in NF responsiveness caused by the ABA treatment.

**Fig. 10.**
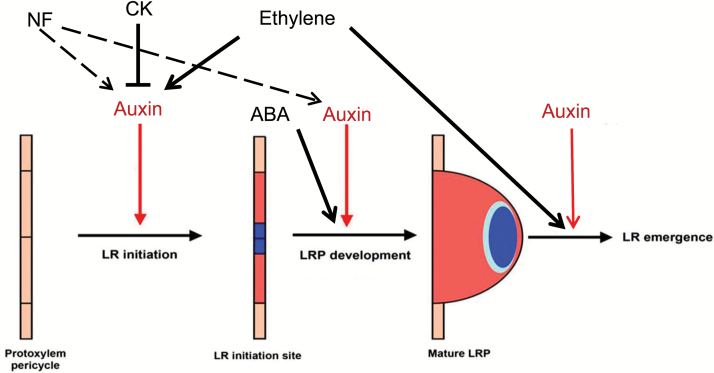
Schematic representation of NFs and phytohormone action on LRF in *Medicago truncatula*. We have shown that NFs interact with auxin and act early on LRF in *M. truncatula*, probably on LR initiation steps, but perhaps also on later stages of LRP development (dashed arrows). Based on the Arabidopsis literature, auxin, cytokinins (CK), and ethylene are known to act on these early steps, but we have shown that NF action on LRF seems independent of the CK receptor MtCRE1 and the ethylene signaling pathway. We have shown in our previous work (Gonzalez *et al.*, 2015) that ABA stimulates intermediate stages of LRF in *M. truncatula* but we did not see any interaction between NFs and ABA on LRF in this study. From the Arabidopsis literature, ethylene and auxin are also known to play roles in later steps of LRF, such as LR emergence (with dose-dependent effects). Blue color highlights zones of auxin maxima.

Figure 10 summarizes the possible effects of NFs and hormonal pathways on LRF in *M. truncatula*.

### *The LRIS is efficient in* M. truncatula *for LRF and NF studies*

Transcriptomic approaches that efficiently target very early stages of LRF are challenging, due to the limited number and the lack of synchronicity of root cells that are committed to LRF programs. Our work shows that the lateral root inducible system (LRIS) originally developed for Arabidopsis (Himanen *et al.*, 2002) and extended to maize (Jansen *et al.*, 2013) can be efficiently used for legume plants such as *M. truncatula*. The same concentration of NPA used for Arabidopsis was efficient to block *M. truncatula* LRF, but a lower concentration of NAA than that initially used for Arabidopsis (10^‒6^ M instead of 10^‒5^ M) proved to be very efficient to restart the LRF program. The order and types of cell layer divisions we observed using the LRIS system were similar to those we described for normal LR development in *M. truncatula* (Herrbach *et al.*, 2014). As expected, the extent of the primary root portion undergoing LRF was significantly increased. We also observed a large number of auxin-responsive genes in our system. By using reciprocal best-hits between the Medicago Mt4.0 genome and the current Arabidopsis genome, we could find candidate orthologs for genes previously described in the Arabidopsis literature using LRIS. For instance, 31 out of the 99 genes found by Vanneste and collaborators to be regulated in a *ARF17ARF19*-dependent manner (Vanneste *et al.*, 2005) were present in our study, including several IAA/AUX (IAA11/IAA13), LBD (LBD16/18/29), and GH3 genes.

Importantly, *M. truncatula* roots retained NF sensitivity at the molecular level in the LRIS. Moreover, we found a large number of well-known NF-responsive marker genes such as *ENOD11* and *PUB1*, and good overlaps with other NF transcriptomic studies (Breakspear *et al.*, 2014; van Zeijl *et al.*, 2015), even if we cannot exclude the possibility that the disturbance of auxin distribution caused by NPA may alter some NF responses. Following NPA treatment, we could not increase the total number of LRs formed with 10^‒6^ M NAA by adding NFs, probably due to the dominant and saturating effect of such a high dose of auxin. However, we could find the synergistic action of NFs and auxin at the transcriptomic level, showing that the NAA treatment did not impair additional NF responsiveness.

### Interactions between NF and auxin signaling pathways

#### The possible molecular basis of these interactions

We found a large proportion (half) of the NF-responsive genes to be also regulated by NAA alone. More importantly, we found genes that responded differently to the combination of the NF+NAA treatment compared to each treatment individually. We could discriminate two major categories in the NF+NAA ‘interaction’ groups: group 1 (1526 genes) corresponds to genes responding to the NF+NAA combination and not significantly to either treatment alone, whereas group 2 (266 genes) comprises genes responding to the NAA treatment (and not to NFs) but whose response was significantly modified by the addition of NFs to NAA. A third, minor category (51 genes) represents genes responding to NFs and not to NAA but whose response was modified in the NF+NAA treatment compared to NFs alone. We confirmed by Q-PCR analysis on the *nfp-2* mutant that the NF+NAA response was dependent on NF perception, emphasizing that these NF+NAA interactions could be *bona fide* symbiotic responses. Both group 1 and group 2 displayed significant enrichments in signaling and metabolic processes, showing that NF treatment can ‘potentiate’ both positive and negative auxin responses of *M. truncatula* roots, especially affecting signaling and metabolic activities. Conversely, auxin can negatively interfere with some NF-responsive genes, as shown in group 3, or influence the expression of symbiotic receptors, as found in group 1. Given the ancestral existence of auxin signaling (Finet and Jaillais, 2012), we can hypothesis that NF (and maybe more broadly LCO) signaling has impacted on auxin signaling pathways to control symbiotic and development programs.

There are a variety of molecular mechanisms that could explain this interaction between NFs and auxin. For instance, NFs are known to interfere with auxin transport (Mathesius *et al.*, 1998) and also possibly with auxin biosynthesis and homeostasis (Larrainzar *et al.*, 2015). In our first synergistic group, we found auxin biosynthesis (*ASA1*, *TAA1*) and an auxin transporter (*MtLAX1*) gene, whereas a potential auxin-conjugating enzyme (similar to GH3-1, AT2G23170) was found in group 2. However, several *PIN* genes (*MtPIN2/3/6*) or *MtLAX2* and *MtLAX3* were only found to be regulated by NAA. A cytokinin oxydase (*MtCKX2*) and several ethylene biosynthetic (ACS) genes were also found in group 2. Ethylene is known to act on auxin biosynthesis and transport in Arabidopsis (Muday *et al.*, 2012). As CKs are known to counteract the action of auxin, expression of CK degradation enzymes could modify the sensitivity of the root tissues to auxin. We could not find any NF or synergistic regulation of auxin receptors such as *TIR1* or *AFB* genes, but we found a few *IAA/AUX* genes in group 1 and one in group 2. Altogether, these data suggest that NFs can interfere with both auxin signaling and biosynthesis or conjugation, but possibly also through crosstalk with other hormonal pathways, as recently shown during nodulation (Larrainzar *et al.*, 2015).

#### The biological relevance of this molecular interaction

We found a clear synergistic effect of NFs and NAA on LRF in *M. truncatula*. A closer look at the differentially expressed genes (DEGs) found in the first two synergistic categories reveals functions that can be linked to LRF, but also highlights crosstalk between NF and auxin pathways for several general biological processes. For instance, both groups displayed functional categories corresponding to metabolic pathways such as phenylpropanoid biosynthesis. This pathway comprises a variety of functions, ranging from cell wall modification to production of defense compounds. Along the same lines, we found ‘biotic stress’ functions, encompassing peroxidase genes and a large number of signaling genes encoding TIR-NBS-LRR and NB-ARC domain disease resistance proteins, largely down-regulated in group 1. These data are in accordance with both auxin and NFs regulating cell wall plasticity and defense mechanisms (Liang *et al.*, 2014; Naseem *et al.*, 2015). Interestingly, cell wall plasticity is important in many processes, including nodulation and LRF (Xie *et al.*, 2012; Lewis *et al.*, 2013). Along the same lines, group 1 up-regulated genes were enriched in developmental processes linked to pollen or root hair development, where auxin is implicated but whose processes could also be relevant for rhizobium infection (Breakspear *et al.*, 2014).

Functional categories enriched in the first synergistic group encompassed transcription function categories such as histones and NF-Y transcription factors, probably linked to cell cycle activation. Different RNA polymerase subunits and elongation factors were also present. This is in accordance with the mitotic and transcriptional regulation activities of both auxin and NFs. This suggests that the combination of NFs and NAA was more efficient to restart the cell cycle and activate transcriptional machinery genes. Interestingly, many ‘protein degradation processes’ such as ubiquitin and proteasome pathways were enriched in down-regulated genes in group 1. Indeed, auxin is well known for its connection to the proteasome degradation pathway (Černý *et al.*, 2016), and E3 ligases/SINA genes are also emerging as important regulators of nodulation (Hervé *et al.*, 2011).

Many genes from the first two synergistic categories could be found to be co-regulated during nodulation using the LegumeGRN and MtGeneAtlas tools from the Noble Foundation (Benedito *et al.*, 2008; Wang *et al.*, 2013) (data not shown). Nodulation, and especially nodule organogenesis, is known to involve auxin and LR-related genes (Breakspear *et al.*, 2014; Larrainzar *et al.*, 2015). We found LBD16 and PLT3 genes to be regulated both by NFs and NAA in our dataset, with a dominant auxin effect. Recently, MtPLT3 was shown to be involved in nodule meristem maintenance (Franssen *et al.*, 2015). Interestingly, the ortholog of one of our group 1 genes, *MtCKX2*, was recently shown to be involved in nodulation in lotus, but it has no impact on LR density (Reid *et al.*, 2016).

Finally, among the 48 NF-responsive genes whose expression was attenuated in the NF+NAA condition (group 3), eight displayed strong expression in mycorrhizal transcriptomic data from the MtGeneAtlas (Benedito *et al.*, 2008). There is already increasing evidence of auxin being implicated during symbiotic interactions (Breakspear *et al.*, 2014; Etemadi *et al.*, 2014), but our results highlight the extensive crosstalk between auxin and NF (maybe more broadly with LCO) signaling that probably controls nodulation and mycorrhization processes.

### LRF candidate genes from our dataset

Our transcriptomic approach revealed a large interaction between NAA- and NF-regulated genes. The main issue that now remains is to pinpoint the most interesting genes that could be responsible for NF effects on LRF. Given the primordial role of auxin in controlling LRF, genes from group 2 may display the most interesting candidates. Interestingly, group 2 genes with a positive interaction between NFs and auxin were enriched in signaling functions.

One way to find such genes is to look for possible orthologs of well-known LRF regulators identified in Arabidopsis. Among such candidates, ACR4, SLR, or ARF7-like genes were only regulated by NAA and no interaction with NFs was visible in our data. Comparison between our DEGs and Arabidopsis root transcriptomic data (Vanneste *et al.*, 2005; Brady *et al.*, 2007) did not reveal obvious candidates, which may be due to the different root tissue contributions to LRF in *M. truncatula* compared to Arabidopsis (Herrbach *et al.*, 2014). Interestingly, we found that the negative regulator of LRF, *MtCRA2*, was negatively regulated by auxin and more repressed by the combination of NAA+NF, but this was not significant when directly compared to NAA. However, MtIAA7, found in group 2, is an interesting candidate as it is phylogenetically related to AtIAA29, which has been shown to interact with ARF7 in Arabidopsis (Sun *et al.*, 2013). In *M. truncatula*, *MtIAA7* seems to be root-specific and responsive to *S. meliloti* inoculation (Shen *et al.*, 2014).

We are currently assessing *M. truncatula* Nod^–^ mutants affected in components downstream of the CSSP to determine if any of them will still respond to NFs for LRF. Looking at the regulation of candidate genes or applying the same LRIS strategy in such mutants, combined with a tissue-specific transcriptomic approach, should help us to further dissect the molecular effects of NFs on LRF.

## Supplementary data

Supplementary data are available at *JXB* online.

Figure S1. LR density of wild-type seedlings after 13 d of culture on different ACC concentrations.

Figure S2. LR density of *Mtskl* and wild-type seedlings after 13 d of culture.

Figure S3. LRF of *Mtskl* seedlings in response to ACC.

Figure S4. Primary root growth of *Mtskl* seedlings in response to NF treatment.

Figure S5. Non-emerged *Mtskl* LRP in response to NF treatment.

Figure S6. Primary root length of *Mtcre-1* seedlings and its wild-type sibling in response to NF treatment. 

Figure S7. Dose–response effects of the auxin analog 1-naphthaleneacetic acid (NAA) on the mean numbers of LRs and the primary root length of wild-type plants in the absence or presence of NFs.

Figure S8. NimbleGen array validation by high-throughput Q-PCR.

Figure S9. Principal component analysis of the 60 genes tested by Q-PCR after 10 h of treatment in the wild-type or the *nfp-2* genetic background.

Figure S10. Mapman representation of the ‘regulation overview’ and ‘biotic stress’ categories for the genes in the first synergistic group.

Figure S11. Mapman representation of the ‘regulation overview’ and ‘biotic stress’ categories for the genes in the second synergistic group.

## Data deposition

The following tables are available at the Dryad Digital Repository: http://dx.doi.org/10.5061/dryad.s43c7.

Table S1. Primer list for Q-PCR.

Table S2. List of genes differentially regulated by NAA.

Table S3. List of genes differentially regulated by NFs.

Table S4. List of genes differentially regulated by NAA+NF compared to control conditions.

Table S5. List of genes from the first synergistic group.

Table S6. List of genes differentially regulated by NAA+NF compared to NAA.

Table S7. List of genes from the second synergistic group.

Table S8. List of genes with a potential hormonal or LRF function.

Table S9. List of genes from the third synergistic group.

## Supplementary Material

supplementary_figures_S1_S11

## Abbreviations:

LR: lateral root
LRP: lateral root primordia
CSSP: common symbiosis signaling pathway
NF: Nod factor.
