# Supplementary material for: Nod factors potentiate auxin signaling for transcriptional regulation and lateral root formation in Medicago truncatula
Source: J Exp Bot. 2017 Jan 10;68(3):569–83. doi: 10.1093/jxb/erw474 (PMC6055581; doi:10.1093/jxb/erw474)
Supplement: supplementary_figures_S1_S11 [file erw474_suppl_supplementary_figures_s1_s11.pdf]

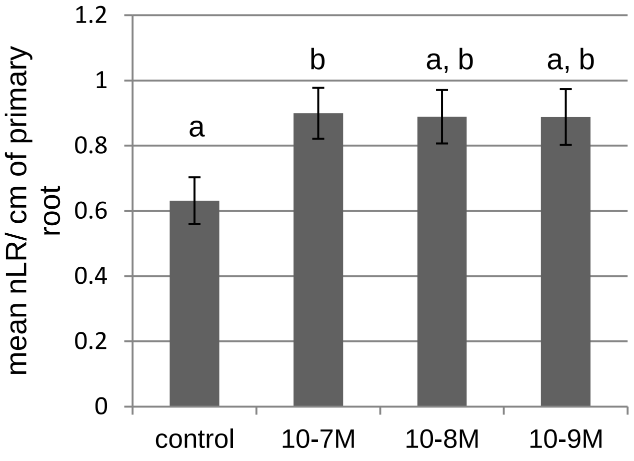

Figure S1. LR density of wild type seedlings after 13 days of culture on different ACC concentrations

Mean number of emerged lateral roots (LR) per cm of primary root after 13 days of growth for A17 plants grown on M-medium containing either no (control with only water, as ACC was diluted in water), 10^{-7} M, 10^{-8} M or 10^{-9} M 1-Aminocyclopropane-I-Carboxylic Acid (ACC). Error bars represent the standard error of the mean (sem). Mean of 3 independent experiments. Superscript letters correspond to significant different groups following a Kruskal-Wallis with post-hoc VanWaerden test (with a Benjamini Hodchberg correction), with a p-value<0.05.

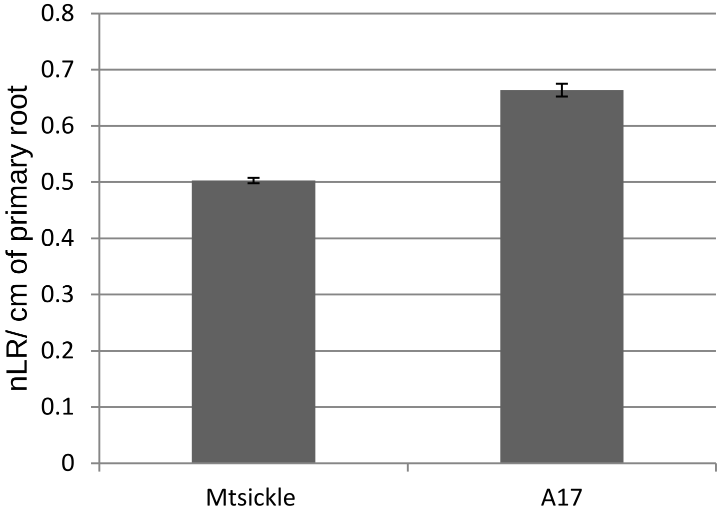

Figure S2. LR density of *Mtskl* and wild type seedlings after 13 days of culture
Mean number of emerged lateral roots (LR) per cm of primary root after 13 days of growth for *Mtskl* and A17 plants grown on M-medium. Error bars represent the standard error of the mean (sem). Mean of 3 independent experiments, Wilcoxon rank sum test not significant.

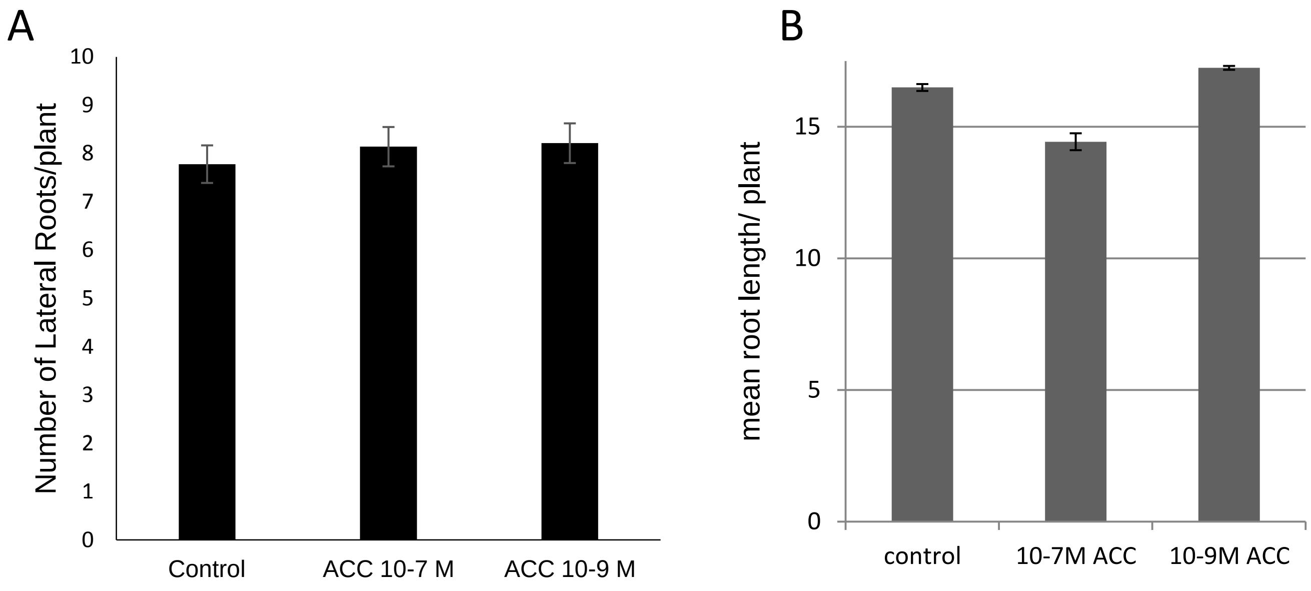

Figure S3. LRF of *Mtskl* seedlings in response to ACC

Mean number of lateral roots (A) and primary root length (B) of *Mtskl* seedlings grown for 13 days on M-medium with 10^{-7} M or 10^{-9} M ACC. The data represents 45 to 53 plants from 3 independent experiments. Error bars represent the standard error of the mean (sem). No significant difference was observed using a non parametric Kruskal-Wallis test between the control and ACC treated plants.

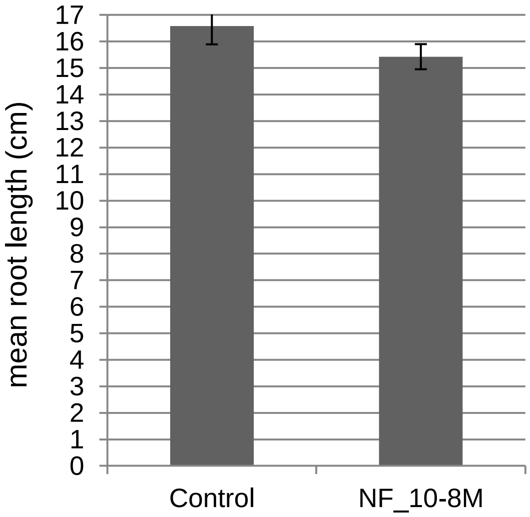

Figure S4. Primary root growth of *Mtskl* seedlings in response to NF treatment
Mean root length of *Mtskl* seedlings grown for 13 days on M-medium in control condition of with 10^{-8} M NF. The data represents 45 to 53 plants from 3 independent experiments. Error bars represent the standard error of the mean (sem). No significant difference was observed using a non parametric Kruskal-Wallis test between the control and NF treated plants.

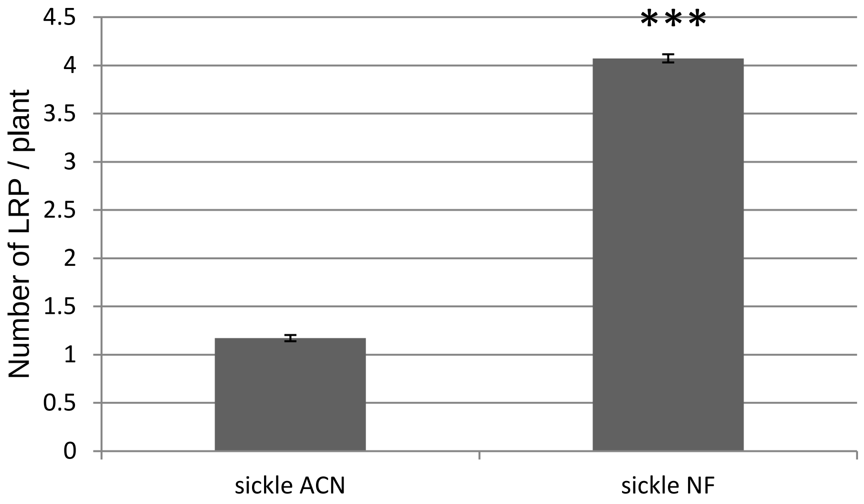

Figure S5. Non emerged *Mtskl* LRP in response to NF treatment

Mean number of non emerged LRP after 13 days of growth of *Mtskl* plantlets treated (NF) or not (ACN) with 10^{-8} M NF. Error bars represent the standard error of the mean (sem). Wilcoxon rank sum test highly significant ($p<0.001$).

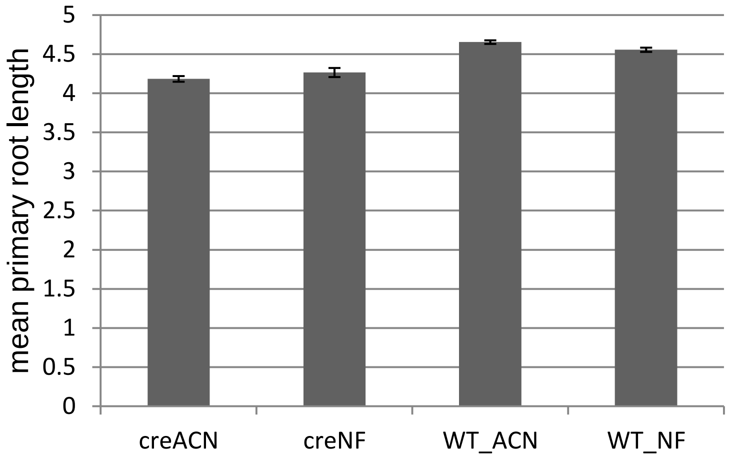

<u>Figure S6. Primary root length of *Mtcre-1* seedlings and its wild type sibling in response to NF treatment</u>

Mean root length of *Mtcre1-1* (cre1-1) and its wild type sibling (WT) after 8 days on a growth medium containing 10^{-8} M NF (NF) or the same quantity of the acetonitrile (ACN) solvent. Results represent the mean primary root length in 2 independent experiments, with 40 seedlings each. Error bars represent the standard error of the mean (sem). No statistically significant difference could be found using a Kruskal-Wallis non parametric test.

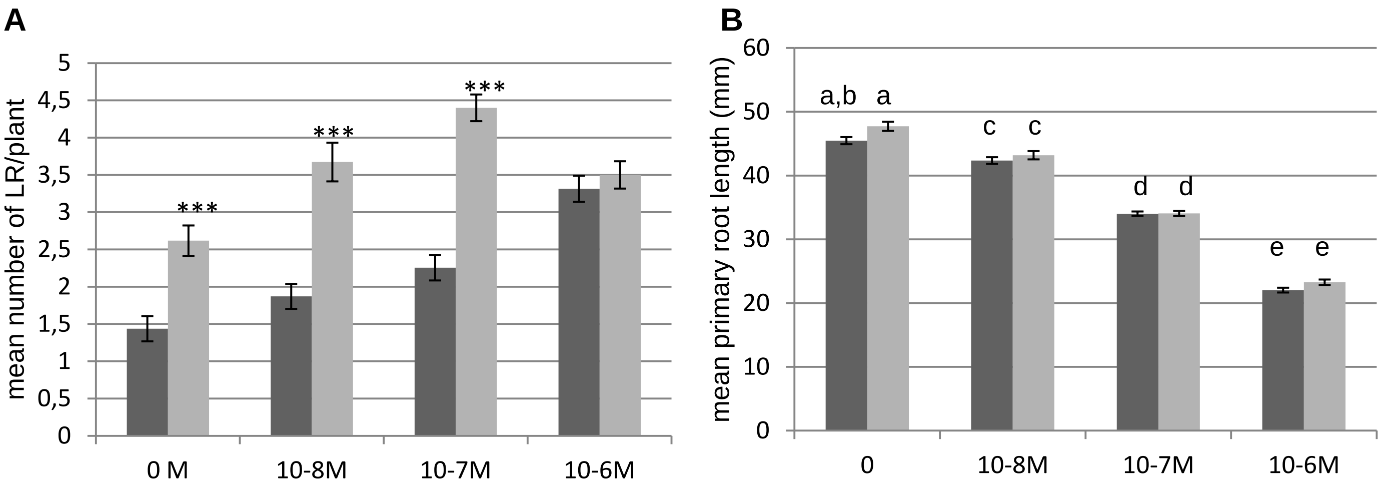

Figure S7. Dose response effect of the auxin analog 1-Naphthaleneacetic acid (NAA) on the mean number of LR and the primary root length of wild type (A17) plants in the absence or presence of NFs.

Results represent the mean number of emerged LR (A) or mean primary root length (B) observed for 38 to 55 plantlets for each treatment in absence (dark grey) or presence (light grey) of 10^{-8} M NF. Error bars represent the standard error of the mean (sem). *** in A mark significant differences following a non parametric Kruskal-Wallis pairwise test ($p<0.001$). In B superscript letters correspond to significantly different groups following a Kruskal-Wallis and post-hoc van der Waerden normal scores test for multiple comparisons (with a Benjamini Hodchberg correction) ($p<0.05$)

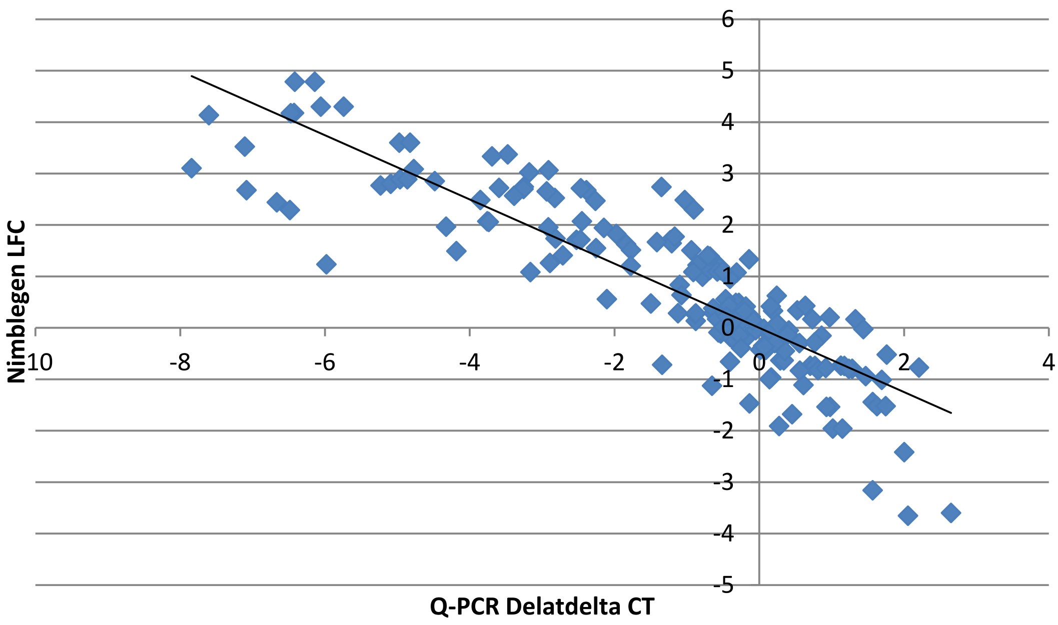

<u>Figure S8. NimbleGen array validation by high- throughput Q-PCR</u>

60 genes in 3 independent repetitions and 3 treatments (NF, NAA, NAA+NF) were tested by BioMark high-throughput Q-PCR. Log2 fold change (LFC) and delta(delta)Ct are calculated in comparison to the non treated corresponding control.
R (Pearson correlation coefficient) = -0.87

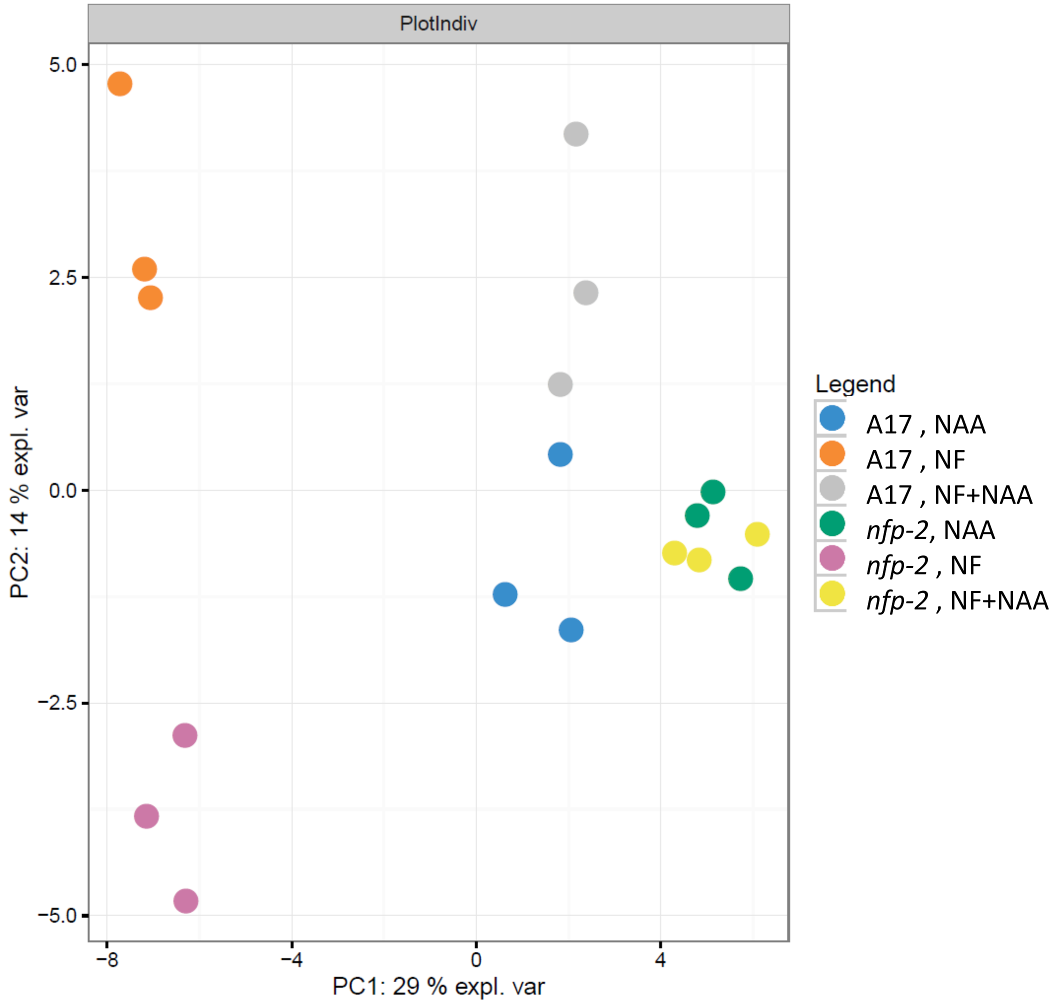

<u>Figure S9. Principal Component Analysis (PCA) of the 60 genes tested by Q-PCR after 10h of treatment in A17 or *nfp-2* genetic background</u>

PCA analysis was done on the expression ratio of 60 genes tested by Q-PCR after 10h of the different treatments: NAA (10^{-6} M NAA), NF (10^{-7} M NF) or NF+NAA in the wild type (A17) or *nfp-2* mutant background.Three biological replicates were obtained for each genotype and treatment and each color dot represents the values of the gene expressions in one treatment and one replicate (Orange: A17, NF treatment; Blue: A17, NAA treatment; Grey: A17, NF+NAA treatment;Pink: *nfp-2*, NF treatment; Green: *nfp-2*, NAA treatment; Yellow: *nfp-2*, NF+NAA treatment)

We can observe that treatment is the major effect for A17, with NF alone (orange) being very different from NAA (blue) and NAA+NF (grey). The NF+NAA effect (grey) is separated from NAA alone (blue) in A17 but not in *nfp-2*, where both treatments overlap (yellow and green).

A

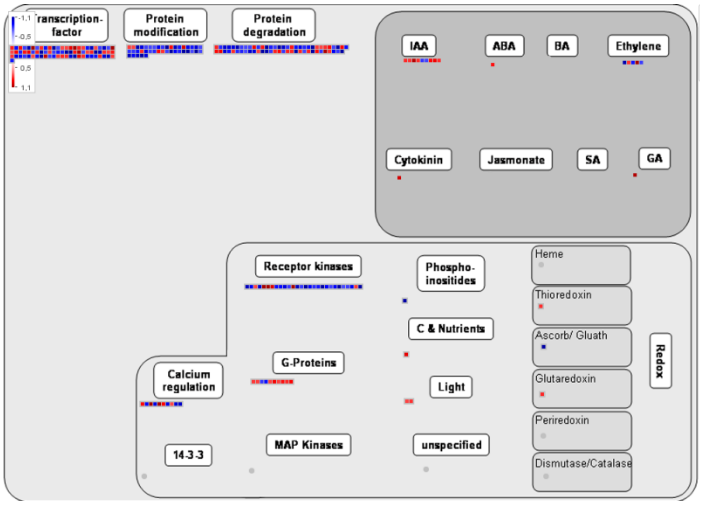

B

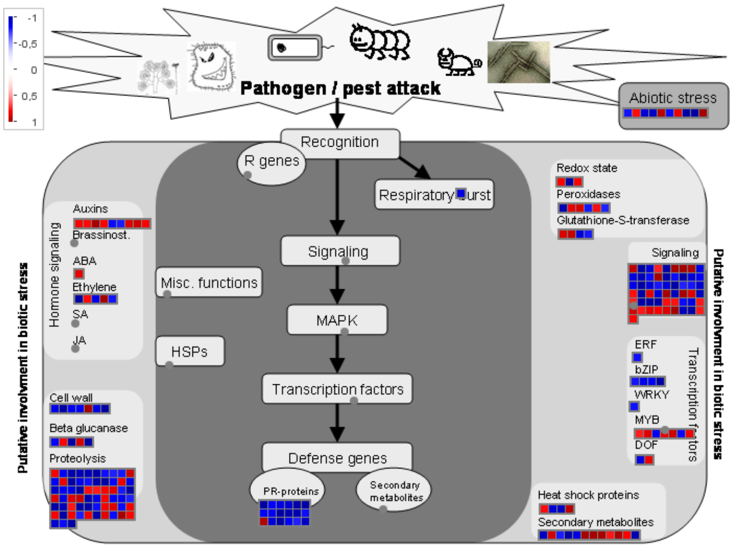

Figure S10. Mapman representation of the "regulation overview " and "biotic stress" categories for the genes in the first synergistic group

(A) Regulation overview representation of up and down-regulated genes in the first synergistic group. 252 genes could be mapped to this scheme.

(B) Biotic stress representation of up and down-regulated genes in the first synergistic group. 218 genes could be mapped to this scheme.

Blue color represents down regulated genes and red color upregulated genes (the color scale is a Log2 fold scale). IAA= auxin, BA= brassinosteroids, SA=salicylic acid, GA= Gibberellins.

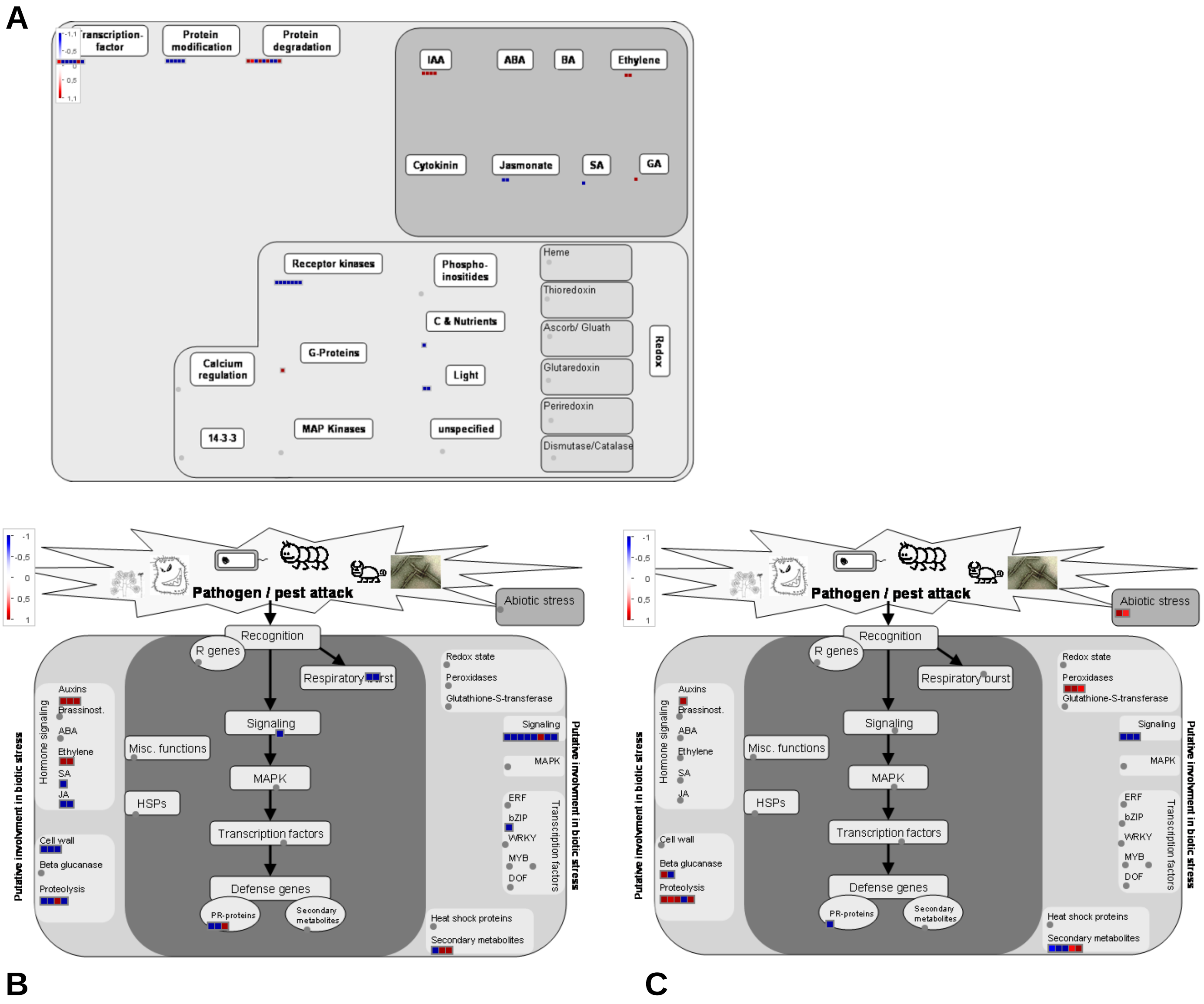

Figure S11. Mapman representation of the "regulation overview " and "biotic stress" categories for the genes in the second synergistic group

(A) Regulation overview representation of genes in the second synergistic group. 42 genes could be mapped to this scheme.

(B) Biotic stress representation of genes in the second synergistic group where NFs enhance NAA regulation. 33 genes could be mapped to this scheme.

(C) Biotic stress representation of genes in the second synergistic group where NFs antagonize NAA regulation. 22 genes could be mapped to this scheme

Blue color represents down regulated genes and red color upregulated genes (the color scale is a Log2 fold scale). IAA= auxin, BA= brassinosteroids, SA=salicylic acid, GA= Gibberellins.
